# Muscle Co-Activation around the Knee during Different Walking Speeds in Healthy Females

**DOI:** 10.3390/s21030677

**Published:** 2021-01-20

**Authors:** Abdel-Rahman Akl, Pedro Gonçalves, Pedro Fonseca, Amr Hassan, João Paulo Vilas-Boas, Filipe Conceição

**Affiliations:** 1 Faculty of Physical Education-Abo Qir, Alexandria University, Alexandria 21913, Egypt; 2 Porto Biomechanics Laboratory (LABIOMEP-UP), Faculty of Sport (CIFI2D), University of Porto, 4099-002 Porto, Portugal; pgoncalves@fade.up.pt (P.G.); pedro.labiomep@fade.up.pt (P.F.); jpvb@fade.up.pt (J.P.V.-B.); filipe@fade.up.pt (F.C.); 3 Department of Sports Training, Faculty of Sports Education, Mansoura University, Mansoura 35516, Egypt; amrahh@mans.edu.eg

**Keywords:** biomechanics, agonist and antagonist muscle activation, knee, injury prevention, EMG

## Abstract

The purpose of this study was to examine the changes in co-activation around the knee joint during different walking speeds in healthy females using the co-activation index. Ten healthy females (age: 21.20 ± 7.21 years, height: 164.00 ± 4.00 cm, mass: 60.60 ± 4.99 kg) participated in this study and performed three walking speeds (slow, normal, and fast). A Qualisys 11-camera motion analysis system sampling at a frequency of 200 Hz was synchronized with a Trigno EMG Wireless system operating at a 2000 Hz sampling frequency. A significant decrease in the co-activation index of thigh muscles was observed between the slow and fast, and between the normal and fast, walking speeds during all walking phases. A non-significant difference was observed between the slow and normal walking speeds during most walking phases, except the second double support phase, during which the difference was significant. A negative relationship was found between walking speed and the co-activation index of thigh muscles in all speeds during walking phases: first double support (r = −0.3386, *p* < 0.001), single support (r = −0.2144, *p* < 0.01), second double support (r = −0.4949, *p* < 0.001), and Swing (r = −0.1639, *p* < 0.05). In conclusion, the results indicated high variability of thigh muscle co-activation in healthy females during the different walking speeds, and a decrease in the co-activation of the thigh muscles with the increase of speed.

## 1. Introduction

Walking is a critical movement for human life, since it allows the human to move between places and perform tasks required for their life. The loss or decrease of walking or running ability represents a severe obstruction to a healthy lifestyle. Moreover, walking speed affects elements of walking kinematics, ground reaction forces, and muscle activity. The muscle activity is an important mechanism that is required for walking stability, especially at different walking speeds [1]. Hof et al. (2002) examined electromyographic (EMG) patterns at a variety of speeds and found considerable changes with speed [2]. den Otter et al. (2004) observed systematic changes in EMG activity between different walking speeds (normal to very slow). Tirosh et al. (2013) examined the variability of muscle activation in children between 7 and 16 years at different speeds and found that muscle activation patterns in children under 10 years are more variable than older children only when walking either slower or faster than the preferred speed [3].

The muscle activity amplitude increases with walking speed, but this increase may change between agonist and antagonist muscles [4]. Previous studies investigated how muscles provide support and forward progression of the body during walking [5,6,7]. Neptune et al. (2008) analyzed two-dimensional computer simulations of walking at five speeds, but they investigated only the agonist muscles [8]. Only a few studies used the activity of antagonist muscles when assessing human walking [9,10,11,12]. Lee, Kang, and Shin (2015) suggested that the responses of antagonist muscles, which have not received much attention before, could be more sensitive than agonist muscles in identifying minor changes in stability during walking [13].

Larsen et al. (2008) indicated the importance of the knee and ankle joints for requiring adequate strength and power and reported a decreased amount of antagonist co-activation in young females [14]. Especially during complex tasks such as walking, it is necessary to allow for changes in the agonist and antagonist roles around the knee and ankle during the gait cycle [15]. Kitatani et al. (2016) investigated muscle co-activation around the ankle joint, as did several previous studies [16,17,18,19], but they did not include any EMG information for the knee [20], so our study focused on the knee joint.

In addition, the study of changes of muscle co-activation between agonist and antagonist muscle during walking is very important for the clarification of muscular function of the thigh muscles at different walking speeds. The increasing of coordination during walking may result in muscular changes such as increased muscle co-activation [21]. For example, the rectus femoris (RF), as one of the most important of the quadriceps muscles for the knee extension as an agonist muscle, and the biceps femoris (BF), as one of the hamstring muscles as an antagonist muscle, are working together to provide the stability of the knee joint during walking [22,23,24]. In addition, previous studies in walking at different speeds have used a treadmill [11,25,26] or a metronome to impose the walking speeds or were based on the participants’ self-selected speeds [27,28,29,30]. In this study, we chose the second solution since it is close to the participants’ lifestyles.

Therefore, the purpose of this study was to examine the changes in muscle co-activation around the knee joint during different self-selected walking speeds in healthy females using the co-activation index. We hypothesized that (1) muscle co-activation around the knee would alter during different self-selected walking speeds, and (2) muscle co-activation would decrease when walking speeds increased.

## 2. Materials and Methods

### 2.1. Subjects

Ten healthy adult females (age: 21.20 ± 7.21 years, height: 164.00 ± 4.00 cm, mass: 60.60 ± 4.99 kg) volunteered to take part in the study. Subjects had no history of neural or musculoskeletal injuries of the lower limbs and were free of pain and injury. All participants were informed of the experimental procedures and objectives and gave written consent in agreement with the Declaration of Helsinki (2013). The study was approved by the Ethical Committee for Human Research of the hosting institution.

### 2.2. Experiment Protocol

**Figure 1:**
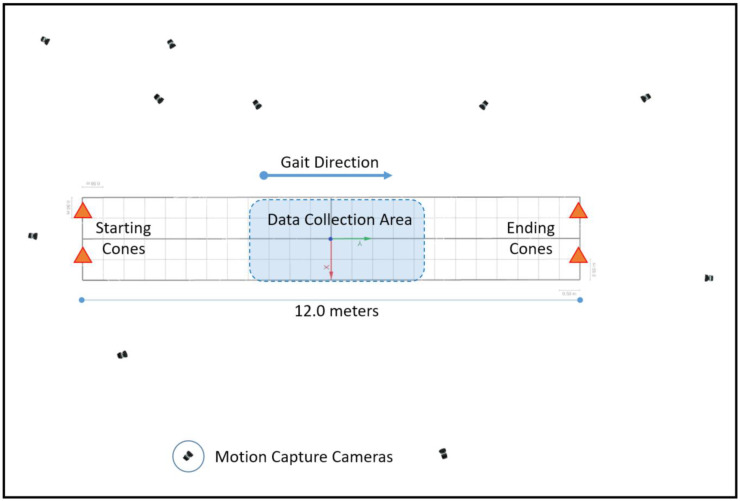
Data collection area for the different walking speeds, camera set-up around the court, and movement direction.

A Qualisys 11-camera motion analysis system (Qualisys AB, Gothenburg, Sweden) operating at a frequency of 200 Hz was used to capture three-dimensional kinematics data of walking at different speeds (Figure 1) and segmentation of the gait cycle. Participants were required to wear short and tight shorts in order to facilitate the markers’ placement and reduce motion artifacts due to the shorts’ fabric.

**Figure 2:**
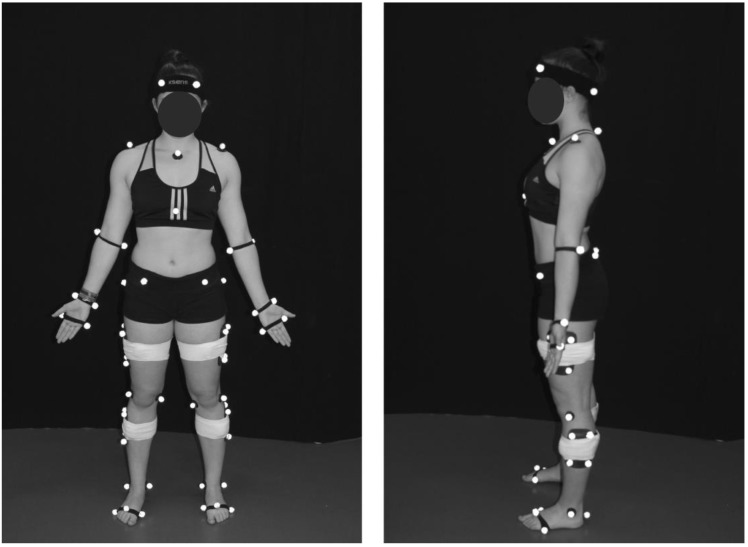
Reflective markers set-up for kinematical modelling.

A modified conventional gait model was used to define body segments with 6 degrees of freedom. Forty-three reflective markers were attached to the body with double-sided adhesive over the following anatomical landmarks: left/right anterior lateral head (located approximately over temple), left/right posterior lateral head (located on the back of the head, roughly in a horizontal plane of the front head markers), left/right acromion, deepest point of incisura jugularis, xiphoid process (sternum), spinous process of the seventh cervical vertebrae, left/right lateral and medial epicondyles of the humerus, left/right radius and ulna styloid processes, left/right dorsum of the 2nd and the 5th metacarpal head, left/right anterior and posterior superior iliac spines, left/right most lateral prominence of the greater trochanter, left/right lateral and medial femur epicondyles, left/right lateral and medial prominence of the malleolus, left/right aspect of the Achilles tendon insertion on the calcaneus, and left/right dorsal margin of the 1st, 2nd, and 5th metatarsal heads. In addition, reflective marker clusters comprising four markers on a rigid base were attached over the lateral mid-thigh and lateral mid-calf of each leg with the help of Superwarp (Fabrifoam, Exton, PA, USA) elastic bandages (Figure 2).

**Figure 3:**
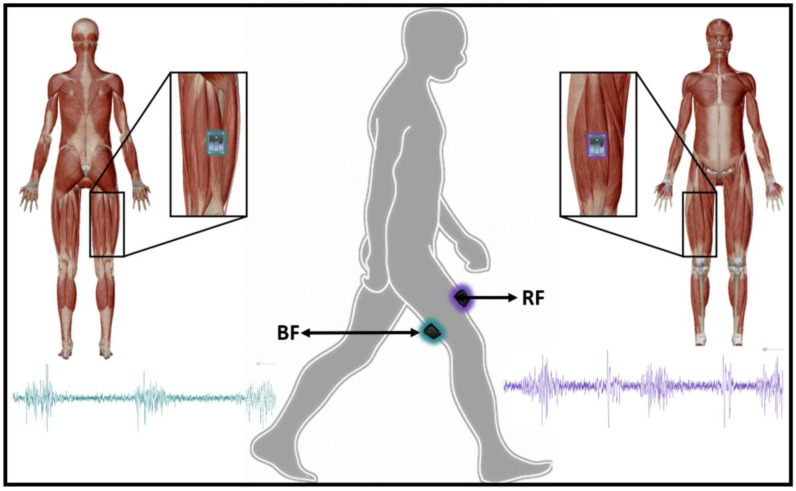
EMG sensors placement on selected muscles.

Surface electromyography (EMG) data from the rectus femoris (RF) and biceps femoris (BF) muscles was recorded during gait by using a Trigno Wireless system (Delsys, Boston, MA, USA) sampling at a rate of 2000 Hz. Each muscle activity was recorded by a wireless sensor containing four silver bar electrodes (size: 5 mm × 1 mm), arranged in two pairs with an inter electrode pair distance of 10 mm [23]. Electrode placement was based on the Surface Electromyography for the Non-Invasive Assessment of Muscles (SENIAM) project recommendations [24] (see Figure 3).

Subjects were asked to perform several straight walking trials over a 12-m walkway at three different familiar speeds: slow, normal, and fast, walking characteristics described in results. The normal walking speed was defined by asking the subject to walk normally according to the subject’s self-regulated speed. For the slow walking speed, participants were instructed to decrease the speed of normal walking about 15–20% and keep the slow speed during the entire walkway. For the fast walking speed, participants were instructed to increase their speed about 15–20% from normal and keep the fast speed during the entire walkway. A distance of at least three strides before and after reaching the force plates was assured, and a single selected stride over the force plates was analyzed for each trial. A total of 220 trials were analyzed for all speeds; 73 trials for slow, 73 for normal, and 74 for fast walking were fit for further data processing. Each participant performed five to eight valid walking trials according to the above-mentioned instructions. The slow and faster trials that were out of range (15–20%) of normal walking speed were ignored during data processing.

### 2.3. Data Processing

**Figure 4:**
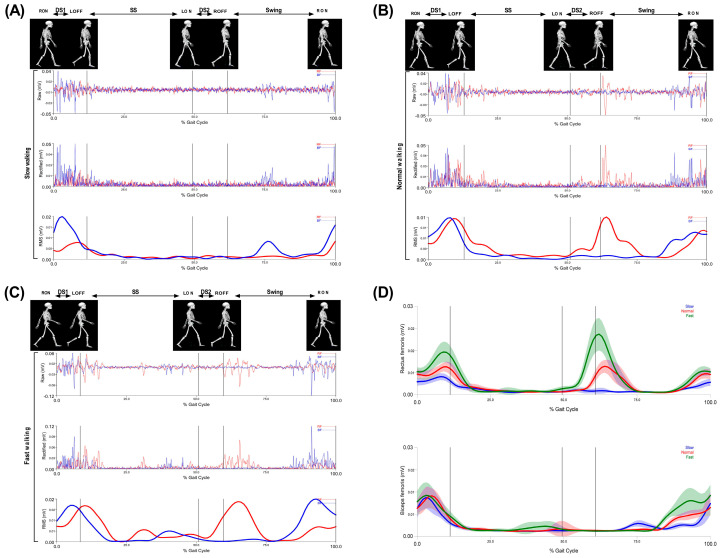
Walking phases: first double support (DS1), single support (SS), second double support (DS2), and swing of the rectus femoris (RF) and biceps femoris (BF). First raw signal; EMG raw data, second raw signal; EMG rectified data and third raw signal; and EMG RMS during (**A**) slow walking, (**B**) normal walking, and (**C**) fast walking. (**D**) Muscle activity amplitude of the three speeds of rectus femoris and biceps femoris.

The collected data were digitized using Qualisys Track Manager Software (Qualisys, Inc., Gothenburg, Sweden) and further processed with the EMG data in Visual 3D software (C-Motion, Germantown, MD, USA). Raw EMG data were filtered using a low band-pass Butterworth filter with a cut-off frequency of 500 Hz and a high-pass Butterworth filter with a cut-off at 20 Hz. The signals were preprocessed using full-wave rectification, then lowpass filtered at 10 Hz, and a linear envelope was obtained using the root mean square (RMS) approach with a window size of 100 ms [31,32], which included normalization of the amplitudes of the EMG signals (Figure 4) [33,34,35].

### 2.4. Muscle Co-Activation

The co-activation index (*CoI*) was the method used for estimating the muscle co-activation of the thigh muscles (RF/BF) during the different walking speeds in this study. The *CoI* was calculated separately for the walking phases: the first double support (DS1), the single support (SS), the second double support (DS2), and the swing phase (Swing), respectively [36,37,38].

The *CoI* was calculated by using the following equation (Equation (1)), which is based on the function of the two muscles during movement, considering that the *BF* is generally the antagonist muscle and the *RF* is the agonist muscle for the knee movement [34,39].

(1) $$CoI=\frac{\int_{t_{1}}^{t_{2}}EMG_{BF}\left(t\right)\;dt}{\int_{t_{1}}^{t_{2}}[EMG_{RF}+EMG_{BF}]\left(t\right)\;dt}\;\times \;100$$

where *t*_1_ and *t*_2_ are the beginning and end of the support phase, *EMG_BF_* the activity of the biceps femoris muscle, and *EMG_RF_* the activity of the rectus femoris muscle for the DS1, SS, DS2, and Swing phases, separately.

According to this method, the *CoI* provides a relative measure of *BF* as a co-activation contribution to the total activation of the *RF* and *BF* during the task [34,39].

### 2.5. Statistical Analysis

The normality of the data was analyzed using Shapiro–Wilk tests and all data were found to be suitable for parametric analysis. Descriptive statistics were reported as means and standard deviations. Analysis of variance (ANOVA) with the Tukey honest significant difference (HSD) post hoc test was used to compare averaged means of muscle co-activation around the knee (CoI RF-BF) between the three walking speeds, during walking sub-phases DS1, SS, DS2, and Swing. In addition, Pearson correlations were used to determine the relationship between walking speeds and CoI RF-BF. All statistical analyses were performed using IBM SPSS software Statistics v21 (IBM^®^ Corporation, Armonk, NY, USA).

## 3. Results

### 3.1. Walking Characteristics

**Figure 5:**
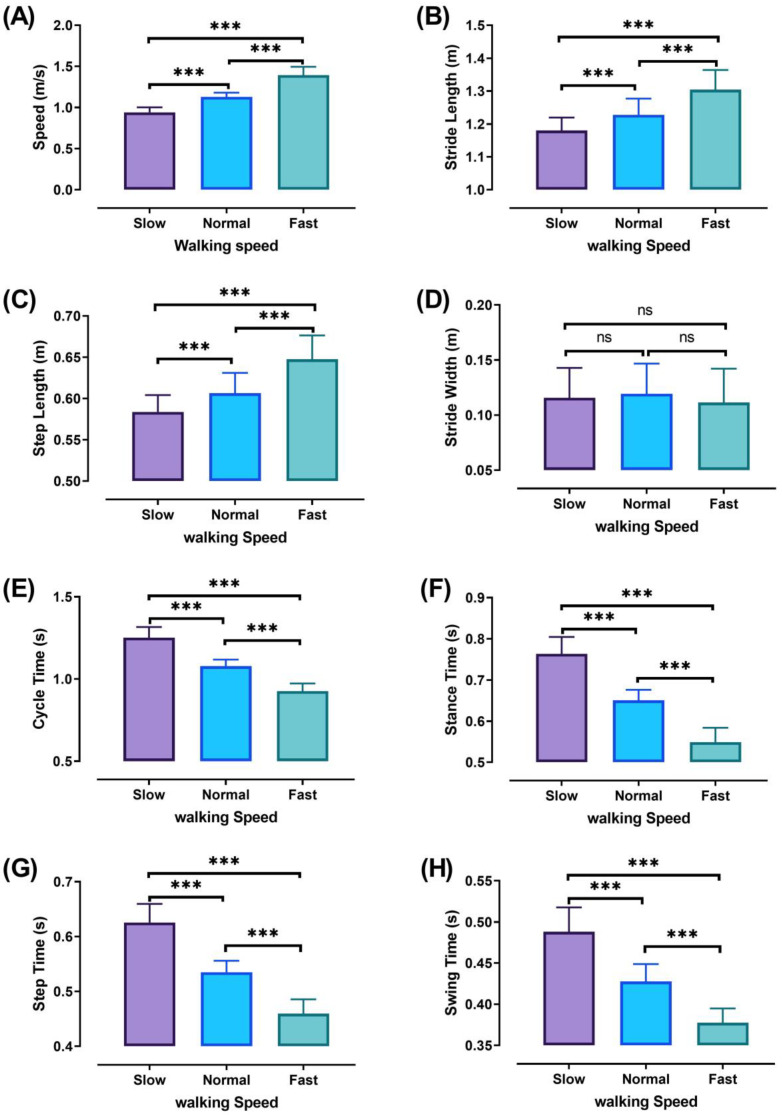
Walking characteristics at different speeds (Slow, Normal, and Fast). walking speed (**A**), stride length (**B**), step length (**C**), stride width (**D**), cycle time (**E**), stance time (**F**), step time (**G**), and swing time (**H**). ANOVA results showing the differences of walking characteristics during walking speed (Slow, Normal, and Fast). Asterisk signs above the line represent significant differences between walking speeds: (***) indicates *p* ˂ 0.001, and (ns) indicates non-significant.

The three walking speeds (slow, normal, and fast) were performed at significantly different percentages (*p* ˂ 0.001). Walking characteristics are shown in Figure 5 with significant values, except for stride width, which did not show significant differences between the different walking speeds. The different percentages of significant walking characteristics are shown in Table 1.

### 3.2. Thigh Muscles CoI at Different Walking Speeds

**Figure 6:**
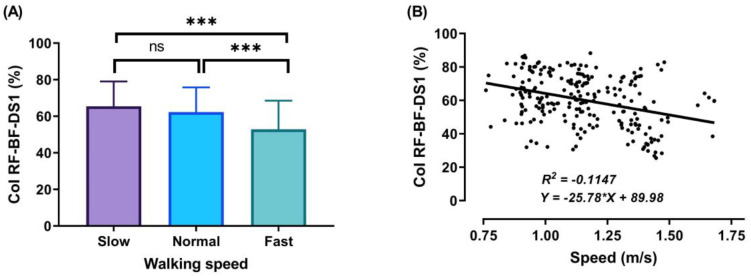
Mean of thigh muscles (RF-BF) co-activation at different walking speeds (slow, normal, and fast) during walking phases: DS1 (**A**). ANOVA results showing the effects of changing in CoI of thigh muscles during walking speed (slow, normal, and fast). Correlation between walking speed and thigh muscles (RF-BF) co-activation during DS1 walking phase (**B**). Asterisk signs above the line represent significant differences between walking speed: (***) indicates *p* ˂ 0.001, and (ns) indicates non-significant.

**Figure 7:**
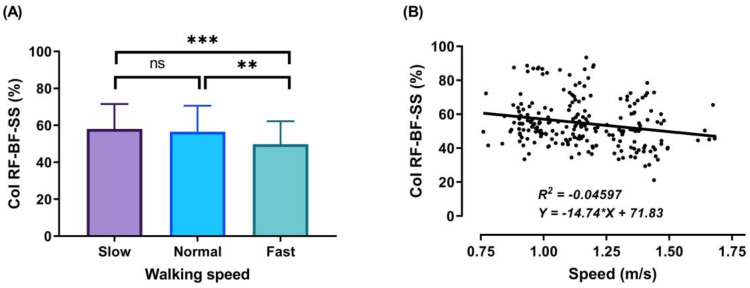
Mean of thigh muscles (RF-BF) co-activation at different walking speeds (slow, normal, and fast) during walking phases: SS (**A**). ANOVA results showing the effects of changing in CoI of thigh muscles during walking speed (slow, normal, and fast). Correlation between walking speed and thigh muscles (RF-BF) co-activation during SS walking phase (**B**). Asterisk signs above the line represent significant differences between walking speed: (***) indicates *p* ˂ 0.001, (**) indicates *p* ˂ 0.01, and (ns) indicates non-significant.

**Figure 8:**
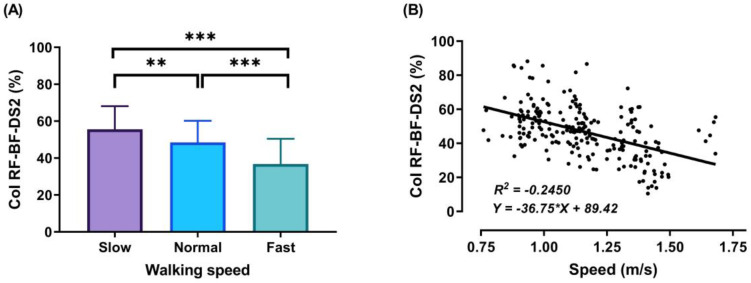
Mean of thigh muscles (RF-BF) co-activation at different walking speeds (slow, normal, and fast) during walking phases: DS2 (**A**). ANOVA results showing the effects of changing in CoI of thigh muscles during walking speed (slow, normal, and fast). Correlation between walking speed and thigh muscles (RF-BF) co-activation during DS2 walking phase (**B**). Asterisk signs above the line represent significant differences between walking speed: (***) indicates *p* ˂ 0.001, and (**) indicates *p* ˂ 0.01.

During the DS1, SS, and DS2 phases, significant decreases (*p* < 0.001) in CoI of thigh muscles were observed between slow to fast walking (23.88%, 16.72%, and 51.33%, respectively). During DS1 and DS2, significant decreases (*p* < 0.001) in CoI of thigh muscles were observed between normal and fast walking (17.85% and 31.67%, respectively), and during SS, significant decreases (*p* < 0.01) between normal and fast walking (13.67%), as shown in Figure 6, Figure 7 and Figure 8A. For DS2, with the increase of walking speed, a decrease of CoI of thigh muscles (14.93%) was observed between slow and normal walking (*p* < 0.01, see Figure 8A). However, no significant CoI of thigh muscles was observed between slow and normal walking during DS1 or SS, or between all speeds during the Swing phase.

### 3.3. Relationship between Walking Speed and CoI of Thigh Muscles

**Figure 9:**
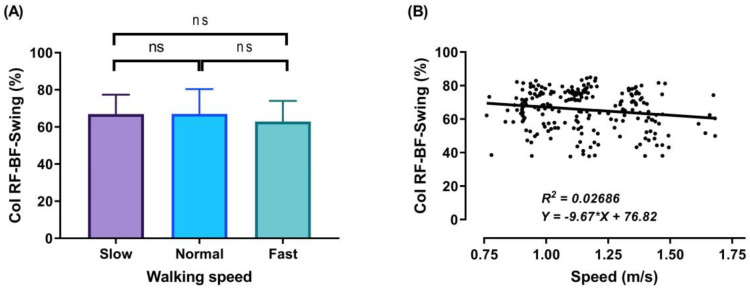
Mean of thigh muscles (RF-BF) co-activation at different walking speeds (slow, normal, and fast) during walking phases: Swing (**A**). ANOVA results showing the effects of changing in CoI of thigh muscles during walking speed (slow, normal, and fast). Correlation between walking speed and thigh muscles (RF-BF) co-activation during Swing walking phase (**B**). Asterisk signs above the line represent significant differences between walking speed: (ns) indicates non-significant.

Pearson correlations analysis showed a negative relationship between walking speed and CoI of thigh muscles in all speeds during walking phases DS1 (r = −0.423, *p* < 0.01), SS (r = −0.285, *p* < 0.01), DS2 (r = −0.573, *p* < 0.01), and Swing (r = −0.214, *p* < 0.01), as shown in Figure 6, Figure 7, Figure 8 and Figure 9B.

## 4. Discussion

To our knowledge, this is the first study to investigate the changes in CoI of thigh muscles as a function of walking speed in healthy females using the co-activation index. The purposes of our study were twofold: to examine the changes in CoI of thigh muscles during three walking speeds (slow, normal, and fast), and also to investigate the relationships between CoI of thigh muscles and walking speed.

Significant differences were observed between all speeds in walking parameters, except the stride width (Figure 5), perhaps because the participants of this study were healthy adult females and had the required ability for preserving balance during walking at different speeds [28,40,41,42,43].

In our study, the walking cycle was divided into four phases (DS1, SS, DS2, and Swing phase) in each of the three different speeds (slow, normal, and fast). During the DS1 phase, the highest magnitude of co-activation was detected (Figure 6A) when the subject moved the leg in front of the body by extending the knee and flexing the hip, with the knee partially flexed and hip moving into extension, allowing weight acceptance and absorbing the shock at the end of this phase. Consequently, to save and control stability during walking involves a combination of distal ankle and proximal hip muscle activation, because it is very important to support and correct the upper body posture [3,44]. Therefore, the RF and BF muscles contracted to achieve stability during this phase. In addition, a decrease was observed in the CoI of thigh muscles with gait speed (for slow walking 65.46 ± 13.55%, normal walking 62.27 ± 13.53%, and fast walking 52.84 ± 15.71%). These results were higher than during the SS and DS2 phases and may be due to the observed changes in the time duration of DS1 when the different walking speeds were compared. Note that the lower limb movements in the DS1 phase start with the extension of the knee and flexion of the hip, and end when the knee is partially flex and the hip starts to extend (Figure 5).

During the SS phase, a significant co-activation was detected (Figure 7A) when the contralateral leg starts leaving the ground. In this phase, the knee and hip of the support leg continue extending, the body weight is exerted over the support leg, and RF and BF act synergistically on the knee joint to control the body movement forward. The average CoI of thigh muscles observed during this phase reveals a decrease with the increase of the speed: slow walking = 58.08 ± 13.45%, normal walking = 56.56 ± 14.03%, and fast walking = 49.76 ± 12.45%.

During the DS2 phase, the third co-activation was observed (Figure 8A) when the participant’s body moves forward, and the leg continues extending. Results showed significant differences in CoI between walking speeds in this phase (slow walking = 55.66 ± 12.47%, normal walking = 48.43 ± 11.77%, and fast walking = 36.78 ± 13.72%). This may be due to the small duration of this particular phase, especially in the fast walking condition, resulting in a small percentage of co-activation.

Finally, during the swing phase, a co-activation was observed (Figure 9A), since in this phase the knee and hip continue flexing to reach the maximum flexion at the midswing, after which the leg moves forward in front of the body by knee extension and the leg slowing down to touch the ground for the next stride. During the swing phase, the average CoI of thigh muscles at the three studied walking speeds was 66.97 ± 10.40%, 67.04 ± 13.32%, and 62.91 ± 11.17% for the slow, normal, and fast walking speeds, respectively.

The outcomes of this study show no significant differences found between slow and normal speed in the CoI of thigh muscles during the DS1, SS, or Swing phases (Figure 6A, Figure 7A and Figure 9A). However, they show instead a significant difference during DS2 (Figure 8A). This may be due to the possibility of thigh muscle contraction during DS2 being affected by the long duration of DS2 in slow walking. Moreover, the results showed that changes in the CoI of thigh muscles between slow and fast speed are significantly greater than between normal and fast conditions during the DS1, SS, DS2, and Swing phases. Therefore, our study indicates that the high activation of antagonist muscles is more important to control gait stability during slow and normal walking than during fast walking. This result is reinforced by the negative relationship noticed between walking speed and CoI of thigh muscles during the DS1, SS, DS2, and Swing phases (Figure 6, Figure 7, Figure 8 and Figure 9B) [27].

Furthermore, our study found a significant negative relationship between walking speed and CoI of thigh muscles during all walking phases: DS1, SS, DS2, and Swing (r = −0.3386, *p* < 0.001; r = −0.2144, *p* < 0.01; r = −0.4949, *p* < 0.001; and r = −0.1639, *p* < 0.05, respectively), as shown in Figure 6, Figure 7, Figure 8 and Figure 9B. This finding suggests that adults probably use a higher activation of antagonist thigh muscles at lower walking speeds to allow enhancing dynamic joint stability. In several previous studies, a high co-activation of antagonist muscles has been observed as a strategy to stiffen the joint and enhance stability [27,45,46]. Thus, increased muscle co-activation may be a necessary change to keep postural control during walking, especially when increasing the duration of the walking cycle by decreasing the walking speed (Figure 5).

## 5. Conclusions

To conclude, the results represent an attempt at quantification of the thigh muscles’ co-activation (RF/BF) in healthy females during walking at different speeds (slow, normal, and fast). Four different co-activations were identified at all the studied walking speeds (slow, normal, and fast), including DS1 (65.46%; 62.27%; 52.84%), SS (58.08%; 56.56%; 49.76%), DS2 (55.66%; 48.43%; 36.78%), and Swing phase (66.97%; 67.04%; 62.91%). However, these always changed inversely with gait speed. This increased co-activation when walking speed is reduced indicates increased requirements of joint stability.

## Figures and Tables

**Table 1:** The different percentages of significant walking characteristics.

Walking Characteristics/Speed	Slow/Normal (%)	Slow/Fast (%)	Normal/Fast (%)
**Speed**	S < N (16.67)	S < F (32.38)	N < F (18.85)
**Cycle time**	S > N (16.09)	S > F (35.04)	N > F (16.33)
**Stance time**	S > N (17.37)	S > F (39.00)	N > F (18.43)
**Step time**	S > N (16.89)	S > F (36.12)	N > F (16.45)
**Swing time**	S > N (14.14)	S > F (29.29)	N > F (13.27)
**Stride Length**	S < N (3.87)	S < F (9.50)	N < F (5.86)
**Step Length**	S < N (3.72)	S < F (9.82)	N < F (6.34)

S: slow walking; N: normal walking; F: fast walking.

## Data Availability

The data presented in this study are available on request from the corresponding author.
